# 

**Published:** 1867-03

**Authors:** 


### Books and Pamphlets Received.

Contributions to the Pathology, Diagnosis, and Treatmeut of Angular Curvature of the Spine. By Benjamin Lee, M. D. Philadelphia: J. B. Lippincott & Co., 1867.

Injuries of the Spine, with an Analysis of nearly four hundred cases. By John Ashhurst, jr., A. M., M. D., Fellow of the College of Physicians of Philadelphia, etc., etc. Philadelphia: J. B. Lippincott & Co. London: Trübner & Co., 1867.

Diphtheria; a Prize Essay. By E. S. Gaillard, M. D., Richmond, Va.

Reduction of Inverted Uteri, by a new method. By Thos. Addis Emmet, M. D., Surgeon in charge of the New York State Woman's Hospital.

Two cases of Œsophagotomy for the removal of foreign bodies, with a history of the operation. By David W. Cheever, M. D., Assistant Professor of Anatomy in Harvard University, etc., etc.

A Series of Articles on Clerical Health, relating to subjects of importance to Pastors and People, written at various leisure moments, some of which were published in 1857. By William M. Cornell, M. D.

The Physical Geography of the North Pacific Ocean, the peculiarities of its circulation, and their relation to the Climate of the Pacific Coast of the United States. By William Henry Doughty, M. D., Augusta, Ga.

Accidental and Congenital Atresia of the Vagina, with a mode of operating for successfully establishing the Canal. By Thomas Addis Emmet, M. D., Surgeon in charge of the New York State Woman's Hospital.

Bulletin of the New York Academy of Medicine. Vivisection; what it is, and what it has accomplished. By John C. Dalton, M. D., Professor of Physiology in the College of Physicians and Surgeons, New York.

Annual Report of the Health Officer of the city of Toledo, for the year 1866.

The Indigestions; or Diseases of the Digestive Organs Functionally Treated. By Thomas King Chambers, Honorary Physician to H. R. H. the Prince of Wales, Consulting Physician and Lecturer on the Practice of Medicine at St. Mary's Hospital, etc., etc. Philadelphia: Henry C. Lea, 1867.

For sale by Theodore Butler, 159 Main street.

Inhalations in the Treatment of Diseases of the Respiratory Passages, particularly as effected by the use of Atomized Fluids. By J. M. Da Costa, M. D., Physician to the Pennsylvania Hospital; Fellow of the College of Physicians; President of the Pathological Society of Philadelphia, etc., etc. Philadelphia: J. B. Lippincott & Co., 1867.

For sale by Breed, Lent & Co.

Forty-fifth Annual Announcement and Catalogue of the Berkshire (Mass.) Medical College.

### American Medical Association.

Office of Permanent Secretary,
Wm. B. Atkinson, M. D.,
215 Spruce street, Philadelphia.

The Eighteenth Annual Meeting of the American Medical Association will be held in Cincinnati, on Tuesday, May 7th, 1867, at 11 o'clock A. M.

The following Committees are expected to report:—

On Quarantine, Dr. Wilson Jewell, Pa., Chairman.

On Ligature of Subclavian Artery, Dr. Willard Parker, N. Y., Chairman.

On Progress of Medical Science, Dr. Jerome C. Smith, N. Y., Chairman.

On the Comparative Value of Life in City and Country, Dr. Edward Jarvis, Mass., Chairman.

On Drainage and Sewerage of Cities, etc., Dr. Wilson Jewell, Pa., Chairman.

On the use of Plaster of Paris in Surgery, Dr. Jas. L. Little, N. Y., Chairman.

On Prize Essays, Dr. F. Donaldson, Md., Chairman.

On Medical Education, Dr. S. D. Gross, Pa., Chairman.

On Medical Literature, Dr. A. C. Post. Chairman.

On Instruction in Medical Colleges, Dr. Nathan S. Davis, Ill., Chairman.

On Rank of Medical Men in the Army, Dr. D. H. Storer, Mass., Chairman.

On Rank of Medical Men in the Navy, Dr. W. M. Wood, U. S. N., Chairman.

On Insanity, Dr. Isaac Ray, R. I., Chairman.
On American Medical Necrology, Dr. C. C. Cox, Md., Chairman.
On the Causes of Epidemics, Dr. Thomas Antisell, D. C.. Chairman.
On Compulsory Vaccination, Dr. A. N. Bell, N. Y., Chairman.
On Leakage of Gas-Pipes, Dr. J. C. Draper, N. Y., Chairman.
On Alcohol and its Relations to Man, Dr. R. W. Dunbar, Md., Chairman.
On the Various Surgical Operations for the Relief of Defective Vision, Dr. M. A. Pallen, Mo., Chairman.
On Local Anæsthesia, Dr. E. Krackowitzer, N. Y., Chairman.
On the Influence upon Vision of the Abnormal Conditions of the Muscular Apparatus of the Eye, Dr. H. D. Noyes, N. Y., Chairman.
On the Comparative Merits of the Different Operations for the Extraction of Vesical Calculi, Dr. B. J. Raphael, N. Y., Chairman.
On the Therapeutics of Inhalation, Dr. J. Solis Cohen, Pa., Chairman.
On the Deleterious Articles used in Dentistry, Dr Augustus Mason, Mass., Chairman.
On Medical Ethics, Dr. Worthington Hooker, Conn., Chairman.
On the Climatology and Epidemics of Maine, Dr. J. C. Weston.
Of New Hampshire, Dr. P. A, Stackpole; Vermont, Dr. Henry Janes; Massachusetts, Dr. Alfred C. Garrett; Rhode Island, Dr. C. W. Parsons; Connecticut, Dr. B. H. Catlin; New York, Dr. E. M. Chapman; New Jersey, Dr. Ezra M. Hunt; Pennsylvania, Dr. D. F. Condie; Delaware, Dr. —— Wood; Maryland, Dr. O. S. Mahon; Georgia, Dr. Juriah Harriss; Missouri, Dr. George Engelman; Alabama, Dr. R. Miller; Texas, Dr. Greensville Dowell; Illinois, Dr. R. C. Hamil; Indiana, Dr. J. F, Hibberd; District of Columbia, Dr. T. Antisell; Iowa, Dr. J. W. H. Baker; Michigan, Dr. Abm. Sager; Ohio, Dr. J. W. Russell.

Secretaries of all medical organizations are requested to forward lists of their delegates as soon as elected, to the Permanent Secretary.

W. B. Atkinson.

### Exchange Journals.

The following Journals are regularly received in exchange:

London Lancet--Editors, J. H. Bennett, M. D., T. Wakely, jr., M. R. C. S. E.

Braithwaite's Retrospect of Practical Medicine and Surgery. New York: W. A. Townsend, 434 Broome street.

The Ophthalmic Review, edited by J. Z. Lawrence and Thomas Winslow, London.

American Journal of the Medical Sciences, edited by Isaac Hays, M. D.

Boston Medical and Surgical Journal, edited by Samuel L. Abbott, M. D. and James C. White, M. D.

The American Journal of Insanity, edited by the officers of the New York State Lunatic Asylum.

The New York Medical Journal.

The Cincinnati Lancet and Observer, edited by Edward B. Stevens, M. D. and John A. Murphy, M. D.

The St. Louis Medical and Surgical Journal, edited by M. L. Linton, M. D. and Frank W. White, M. D.

The Medical Record.

The Chicago Medical Journal.

The Chicago Medical Examiner, edited by N. S. Davis, M. D..

The Medical and Surgical Reporter, edited by S. W. Butler, M. D.

The Cincinnati Journal of Medicine, edited by George C. Blackman, M. D., Theophilus Parvin, M. D. and Roberts Bartholow, M. D.

The Richmond Medical Journal, edited by E. S. Gaillard, M. D. and W. S. Mc-Chesney, M. D.

The Savannah Journal of Medicine, edited by Juria Harris, M. D., J. B. Read, M. D. and J. G. Thomas, M. D.

The Medical Reporter, edited by J. S. B. Alleyne, M. D. and O. F. Potter, M. D.

The Detreit Review of Medicine and Pharmacy, edited by George T. Andrews, M. D., S. T. Duffield, M. D. and E. W. Jenks, M. D.

Atlanta Medical and Surgical Journal, edited by J. G. Westmoreland, M. D.

The Medical and Surgical Monthly, Memphis, Tenn., edited by Frank A. Ramsay, M. D., D. D. Saunders, M. D., E. Mills Willett, M. D. and William H. White, M. D.

Southern Journal of Medical Sciences, edited by E, D. Turner, M. D., D. Warren Brickell, M. D. and C. Beard, M. D.

The Pacific Medical and Surgical Journal and Press, edited by Henry Gibbons, M. D.

The Galveston Medical Journal, edited by Greenvill Dowell, M. D.

The New Orleans Medical Record, a semi-monthly Journal of the Medical Sciences, edited by Bennett Dowler, M. D. and S. R. Chambers, M, D.

The American Journal of Pharmacy, edited by William Proctor, jr.

Medical News and Library, by Henry C. Lea, Philadelphia.

The Druggist's Circular and Chemical Gazette; The Journal of Materia Medica, by Joseph Bates, M. D. and H. A. Tilden.

The Dental Cosmos, edited by J. H. McQuillen, D. D. S. and George J. Zeigler, M. D.

The Atlantic Monthly, published by Ticknor, Fields & Co., Boston.

Godey's Lady's Book, published by Louis A. Godey, Philadelphia.

The Herald of Health.

American Eclectic Medical Review, New York.

Eclectic Medical Journal, Cincinnati, Ohio.

Dental Register, Cincinnati.

The Nation.

Every Saturday, published by Ticknor, Fields & Co., Boston.

Educational Monthly.

